# Club Drugs: Psychotropic Effects and Psychopathological Characteristics of a Sample of Inpatients

**DOI:** 10.3389/fpsyt.2020.00879

**Published:** 2020-08-31

**Authors:** Giovanni Martinotti, Attilio Negri, Stefania Schiavone, Chiara Montemitro, Chiara Vannini, Gaia Baroni, Mauro Pettorruso, Fabio De Giorgio, Raffaele Giorgetti, Valeria Verrastro, Luigia Trabace, Andres Garcia, Ivan Castro, Juan Iglesias Lopez, Cristina Merino Del Villar, Fabrizio Schifano, Massimo di Giannantonio

**Affiliations:** ^1^Department of Neuroscience, Imaging, Clinical Sciences, University G.d’Annunzio Chieti-Pescara, Chieti, Italy; ^2^Department of Pharmacy, Pharmacology and Clinical Science, University of Hertfordshire, Hatfield, United Kingdom; ^3^Postgraduate School of Clinical Pharmacology and Toxicology, University of Milan, Milan, Italy; ^4^Department of Clinical and Experimental Medicine, University of Foggia, Foggia, Italy; ^5^Section of Legal Medicine, Department of Health Care Surveillance and Bioethics, Università Cattolica del Sacro Cuore, Rome, Italy; ^6^Fondazione Policlinico Universitario A. Gemelli IRCCS, Roma, Italy; ^7^Section of Legal Medicine, Department of Excellence SBSP, University “Politecnica delle Marche” of Ancona, Ancona, Italy; ^8^Department of Medical and Surgical Sciences, Magna Graecia University of Catanzaro, Catanzaro, Italy; ^9^Emergency Staff Group, Ibiza, Spain; ^10^Can Misses Hospital, Ibiza, Spain

**Keywords:** club drugs, novel psychoactive substances, psychopathology, psychosis, substance use disorder

## Abstract

**Background:**

Growing evidence supports the possibility of significant psychiatric consequences related to novel and traditional psychoactive substance consumption. The problem of differential diagnosis has hampered research on specific psychopathologies with unclear outcomes. The aim of our study was to report psychiatric and clinical features of subjects admitted to a psychiatric ward in Ibiza, Spain, with a clinical diagnosis of substance abuse or intoxication.

**Methods:**

A survey was administered to a sample of inpatients hospitalized due to psychiatric symptoms related to recent use of psychoactive substances. The questionnaire investigated sociodemographic factors, familiar and personal anamnesis, substance use habits, general and psychopathological features. Urine samples were collected and analyzed in a toxicology laboratory using gas chromatography and mass spectrometry.

**Results:**

A total of 110 patients were included in the study. Most patients (70%) declared multiple substance use, and 33% of patients reported more than two substances; nevertheless, it was possible to identify 17 (15%) depressor users, 44 (40%) stimulant users and 49 (45%) psychodysleptics users. A positive association with a lifetime diagnosis of bipolar disorder was found (two-tailed Fisher’s exact test: p = 0.013). Psychomotor agitation, reference, and paranoid delusions, affective symptoms, consciousness disorders, and aggressiveness represented some of the most frequent symptoms at entry evaluation.

**Conclusions:**

In this study, we described the acute psychiatric presentations related to recreational drug use in subjects on holiday in Ibiza. The use of psychoactive substances was characterized by poly-use of both traditional and novel substances, with several psychopathological consequences. Future research should focus on a better understanding of the psychopathological effects of specific substances, defining signs and symptoms to help make a differential diagnosis and prospectively examine long-term effects.

## Background

Psychoactive substance use and related risks are considered a worldwide major public health issue, involving a variety of health and social consequences that require prompt sanitary policies as well as constantly updated responses from health professionals to promote harm reduction (1). In 2017, an estimated 271 million people worldwide had used psychoactive substances during the previous year—a number 30% higher than that in 2009 (2)—putting substance use disorders (SUDs) among the leading causes of disability worldwide (3). However, although consequences may be dramatic in terms of morbidity, mortality, and psychiatric load, such a widespread phenomenon is often defined as merely an aberrant behavior by modern society.

As shown by recent trends, the extent of problematic drug use is not limited to subjects with SUD or addiction; admissions to specific clinical settings, such as Emergency Rooms (ERs) and psychiatric wards, due to substance-related conditions involve a heterogeneous cohort of users with different motivations of intake. These range from traditional drug users to “psychonauts”, clubgoers, students, athletes, marginalized populations, and individuals with patterns of non-habitual recreational drug consumption (4). Nevertheless, rates of SUD are significantly higher (up to 50%) among psychiatric patients than in the general population (5). In recent years, this rapidly evolving scenario has been further complicated by the rise of novel psychoactive substances (NPS). The United Nations Office on Drugs and Crime reports define NPS as “substances of abuse, either in a pure form or a preparation, that are not controlled by the 1961 Single Convention on Narcotic Drugs or the 1971 Convention on Psychotropic Substances, but which may pose a public health threat” (2). Many of these substances were originally developed as research chemicals, as they often mimic the pharmacological effect of traditional drugs of abuse, such as cannabis or phenethylamines, and they were subsequently repurposed for recreational use. NPS are formulated in a variety of forms, both pure and in preparations with other substances. When smoked, ingested, snorted, or injected they may produce a plethora of psychotropic effects, of which some are not fully described. This, along with their often-unknown toxicological profile, their low traceability and the fast-moving and potentially limitless nature of their online market, initially raised significant concern among health professionals (6–8). Although a great effort has been made in the last decade by the scientific community to raise awareness and gain a better knowledge of such compounds and their related risks (9), the understanding of various features, including patterns of consumption, must be further developed (10).

Due to their diverse nature, various categorizations of NPS have been proposed, according to their origin, chemical structures or pharmacological action, among others. Based on their clinical effects on the central nervous system, NPS can be classified as stimulants, empathogens, entactogens, sedative-hypnotics/anxiolytics, dissociatives, and hallucinogens (11). In 2019, the total number of NPS identified worldwide was over 950, of which the vast majority (almost 800 different compounds) had been notified within the last decade (12). Although an increasing number of studies report the potential acute and chronic health dangers associated with such use, NPS and their clinical effects and related risks are often unknown to both users and health professionals, mainly due to a lack of evidence-based sources of information and to the ever-changing nature of their market (4, 9).

Nevertheless, growing evidence supports the possibility of significant psychiatric and physical consequences related to NPS consumption (13, 14). Recently, researchers have reported that NPS consumption may be associated with the onset of a variety of psychiatric symptoms and conditions, including confusion, paranoid thoughts, auditory and visual hallucinations, dissociation (e.g., derealization and somatopsychic depersonalization), insomnia, chronic cognitive impairment and delusions of reference, persecution, grandeur and jealousy, as well as hypomanic states, aggressiveness and irritability, violence, and suicidal thoughts (15–17). These symptoms are often due to the increased potency of NPS compared to traditional substances, as well as to their action on a number of different neural pathways, including dopamine (DA) and serotonin (5-HT) receptors for psychedelic phenethylamines, tryptamines and synthetic cathinones, cannabinoid (CB) receptors for synthetic cannabinoids, and N-methyl-D-aspartate (NMDA) receptors for some dissociatives (12). Such effects are particularly alarming for compounds that are frequently sold and advertised as natural and safer alternatives to other drugs. For example, observational research on a cohort of 594 synthetic cannabinoids users reported a higher prevalence of psychotic symptoms than in cannabis users (18). Furthermore, the study showed that psychotic disorders were usually more severe in synthetic cannabis users, and patients required higher doses of antipsychotic medications and were hospitalized for longer.

The growing prevalence of novel and traditional psychoactive substances, and their related risks, is further complicated by the phenomenon of “nightlife” and “clubbing” associated with international travels. Holiday periods, particularly in summer, appear to represent a time of risk, excess and experimentation, especially among young people (19). Substance use is a commonly reported habit among festivalgoers and clubgoers in holiday resorts—environments in which hedonistic partying is socially accepted and drugs are typically readily available—which often involve practices such as polysubstance use (i.e., the consumption of two or more compounds simultaneously) (20, 21). Several studies reported the detection in such contexts of a variety of traditional drugs and NPS, such as synthetic cathinones, synthetic cannabinoids, opioids, and naturally derived drugs (e.g., psilocybin, and ayahuasca), by using different screening techniques that ranged from wastewater analysis to self-report surveys (22–24).

Although virtually any psychoactive substance may be used in such an environment for recreational purposes, the National Institute on Drug Abuse (NIDA) includes six compounds among the so-called “club drugs” or “rave drugs” (i.e., drugs commonly consumed during electronic music festivals, dance parties or raves from the 1970s to the present): flunitrazepam, ketamine, LSD, methylenedioxymethamphetamine (MDMA), methamphetamine, and gamma-hydroxybutyrate (GHB) (25). Users, especially young subjects who are often unaware of health-related risks and of the nature of such compounds, including potential contamination and adulteration (26), seek positive effects such as euphoria, improved psychomotor speed, alertness, sociability and talkativeness, amplification of sensory perceptions, alteration of space and time perception, loss of inhibition, increased libido, and improved sexual performance (27–29).

The current dynamic drugs scenario raises enormous concerns for public health at a national and international level. The risks posed by club drugs and related phenomena require adequate training for health professionals, effective harm reduction interventions and updated policies provided by local and supranational regulatory agencies (30). Addressing these challenges may be crucial for guiding the diagnostic and therapeutic options of healthcare professionals, as well as for counteracting related psychiatric and physical risks, such as acute toxicity. For this context, Ibiza and the Balearic Islands, one of the most popular nightlife resorts for summer holidays in Europe, appear as a crucial setting to explore psychopathological issues related to both traditional drugs and NPS. Previous studies available in literature confirmed a higher prevalence of risky behaviors for both residents and tourists in Ibiza, including problematic alcohol and substance use, and their connections with sexual disinhibition, casual sexual relationships and unprotected sex (19, 31–33). Furthermore, anecdotal cases of NPS intoxication have been reported in recent years in the Ibiza drug market; in such a dynamic setting, naive customers are frequently seen by traffickers and dealers as test subjects for trialling new and potentially dangerous compounds for the first time (33).

Our previous reports on this topic investigated substance-related fatalities and provided a first insight of traditional drug- and NPS-induced psychopathological symptoms, particularly regarding aggressiveness (30, 33). The aim of the present study was to analyze patients admitted to the psychiatric ward of the Can Misses Hospital in Ibiza for psychoactive substance intoxication, in order to (a) report the sociodemographic characteristics of the sample, (b) identify which drug is most involved; (c) assess psychopathological features associated with substance use, particularly regarding psychotic symptoms (referring to bipolar or schizophrenic spectrum disorder) and behavioral disturbances.

## Material and Methods

### Patient Recruitment

Subjects admitted to the Can Misses Hospital psychiatry ward during the summer nightclub opening periods between May 2015 and October 2018 were recruited for the study. This sample was derived from a larger sample of 223 subjects transferred to hospital ER from discotheques and other clubs around the island who were exhibiting intoxication and/or manifestations of psychiatric interest. All patients were evaluated according to the DSM-5 diagnostic classification. Inclusion criteria were being aged between 18 and 75 years and reporting the intake of psychoactive substances or more than five units of alcohol over the previous 24 hours. Patients with delirium tremens, epilepsy, liver encephalopathy, dementia and other neurological diseases, severe cardiac failure, diabetes mellitus, severe liver impairment, kidney failure, or neoplastic diseases were excluded after clinical evaluation.

### Data Collection

Demographic (age, gender, family, and nationality) and socioeconomic data (living status, job status, and level of education) were collected in a structured interview administered during hospitalization, after the resolution of intoxication symptoms. The interview investigated recent and past medical and psychiatric history in addition to alcohol and substance use habits (tobacco, caffeine, cannabis, cocaine, and heroin), with a focus on NPS. Among these, recent and lifetime use of synthetic cannabinoids, synthetic cathinones [e.g., mephedrone, methylone, methylenedioxypyrovalerone (MDPV), alpha-pyrrolidinopentiophenone (α-PVP)], amphetamine and methamphetamine, plant-based substances (e.g., ayahuasca, kratom, Salvia divinorum), GHB and GBL (γ-butyrolactone), dissociative substances (e.g., ketamine and methoxetamine), and psychedelics (e.g., LSD, magic mushrooms) was investigated. Misuse of prescription drugs such as benzodiazepines, methylphenidate, and opioid painkillers was also explored. A urine sample was collected upon admission, stored at −30°C and subsequently analyzed at the laboratory of either the Department of Forensic Toxicology of the Università Politecnica delle Marche, Italy, or the National Institute of Toxicology and Forensic Sciences in Barcelona, Spain. Gas chromatography/mass spectrometry (GC/MS) was used to confirm the consumption of psychoactive substances and/or prescription drugs.

### Psychodiagnostic Tests and Analysis

The following psychodiagnostic tests were administered to patients during their hospitalization: Timeline follow-back for psychoactive substances and alcohol (TLFB); Positive and Negative Symptoms Scale (PANSS); Brief Psychiatric Rating Scale (BPRS); Mania Rating Scale (MRS); Hamilton Depression Scale (HAM-D); Hamilton Anxiety Scale (HAM-A); Modified Overt Aggression Scale (MOAS). TLFB was used to identify the main substance of abuse for each patient. The other psychometrics were used to explore different psychopathological aspects, such as depressive or manic symptoms, anxiety, psychosis negative or positive symptoms, somatic disorders, aggressiveness, and suicidality. The choice of psychometric instruments was derived from our previous studies on the topic (4, 33). Patients were divided into three main substance macro-categories according to the TLFB and the results of the urine analysis: Psychostimulants (e.g., cocaine, amphetamines, and synthetic cathinones), Psychodepressors (e.g., opioids, alcohol, and benzodiazepines) and Psychodysleptics (e.g., cannabinoids, psychedelics, dissociatives, empathogens, and entactogens). This classification was derived from our previous reports on the topic (4, 33). According to their pharmacological profiles (11), patients were also allocated to a specific group: Opioids, Stimulants, Empathogens-Entactogens, Psychedelics, Dissociatives, Cannabinoids, and Depressors. Urinalysis was performed in two separate laboratories (to enhance the level of sensitivity and specificity).

### Ethics

Data collection was carried out anonymously and confidentially; all participants received a detailed explanation of the design of the study and written informed consent was systematically obtained from every subject, according to the Declaration of Helsinki. Ethical approval was granted by the University of Hertfordshire Health and Human Sciences ECDA, protocol no. aPHAEC1042(03); by the CEI Illes Balears, protocol no. IB 2561/15 PI (“Estimación y Evaluación de síntomas inducidos por Sustancias y Alcohol”, Dr.a Cristina Merino del Villar, Area de Salud Mental de Eivissa y Formentera); and by the University “G.d’Annunzio” of Chieti-Pescara, no. 7/09 04-2015.

### Data Analysis

Statistical analysis was performed by using IBM SPSS^®^ Statistics software, version 20 and GraphPad 5.0 software for Windows (La Jolla, CA, USA). Independence between substance use and psychiatric diagnosis, as well as between substance use and symptoms at admission, was analyzed using a two-tailed Fisher’s exact test. Correlation between substance categories or groups and psychiatric scales scores was analyzed by linear regression. Data concerning scale scores according to categories and groups of substances were analyzed by one-way analysis of variance (ANOVA), followed by Tukey’s *post hoc* test. For all tests, a p value of <0.05 was considered statistically significant.

## Results

### Sociodemographic Data

A total of 223 subjects showing a condition of intoxication and/or manifestation of psychiatric interest were transferred to the hospital ER from discotheques and other clubs around the island. Of this sample, 110 (49.3%) required subsequent psychiatric hospitalization and were enrolled in the study, whereas 50.7% of the sample only required a 2- to 36-hours stay in the emergency department before a rapid discharge. Sociodemographic information for the sample is reported in **Table 1**.

**Table 1:** Sociodemographic characteristics of the sample.

Characteristic	n	%
**Sample size**	110	100
**Gender** Male Female	76 34	69.1 30.9
**Nationality** European Other Not available (NA)	79 30 1	71.8 27.3 0.9
**Education level** Primary education Secondary education Higher education/undergraduate Higher education/postgraduate NA	9 18 42 19 22	8.2 16.3 38.2 17.3 20.0
**Working status** Worker/professional Student Student and worker Unemployed Retired NA	52 6 3 40 1 8	47.3 5.4 2.7 36.4 0.9 7.3
**Marital status** Single/never married Divorced Married NA	66 21 13 10	60.0 19.1 11.8 9.1
**Living status** With parents With a partner Alone With partner and child/children With friends/flatmates Other NA	29 22 27 13 10 2 7	26.4 20.0 24.5 11.8 9.1 1.8 6.4
**Age subgroups** <30 years old 31–50 years old >50 years old	57 47 6	51.8 42.7 5.5
	Range (years)	Mean (SD)
**Age**	19–63	32.57 (9.15)
**Total years of education**	5–27	12.91 (4.48)

### Categories and Groups of Abused Substances

All the subjects of the sample were diagnosed with substance intoxication at admission. Although the majority of patients declared multiple substance use (n = 77, 70.0%) and 33% of them reported more than two substance use, participants were divided into three macro groups according to their responses to the TLFB test and to the urinalysis in order to identify a category of substances “of choice” for each patient. This allowed to identify 17 (15%) depressors users, 44 (40%) stimulant users and 49 (45%) psychodysleptics users. When asked about lifetime use of specific groups of substances, stimulant use was disclosed by 74 patients and cannabis use by 68 patients. Categories of substances according to the TLFB and urinalysis, as well as prevalence for each substance group, are listed in **Table 2**. Cannabis and cannabinoids (40%) were the most common substances among patients who declared the use of only a single type of drug.

**Table 2:** Categories and groups of abused substances.

Categories	n	%
Psychodysleptics	49	45
Psychostimulants	44	40
Psychodepressors	17	15
**Groups**	n	%
Stimulants	74	32
Cannabinoids	68	29
Depressors	32	14
Empathogens-Entactogens	28	12
Dissociatives	15	6
Opioids	9	4
Psychedelics	7	3

**Figure 1:**
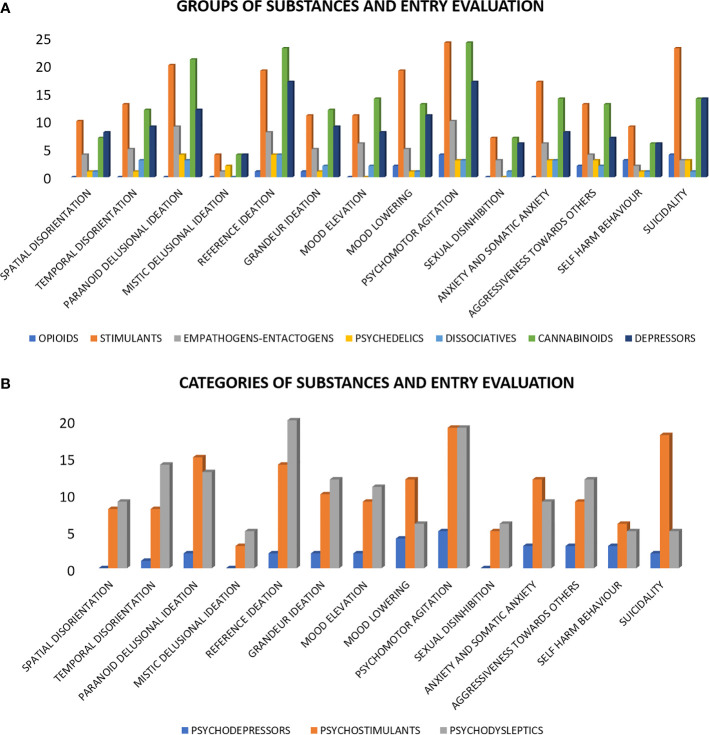
Entry evaluation distribution according to groups and categories of substances. **(A)** Entry evaluation distribution according to the following groups of substances: opioids, stimulants, empathogens-entactogens, psychedelics, dissociatives, cannabinoids, and depressors. **(B)** Entry evaluation distribution according to the following categories of substances: psychodepressors, psychostimulants, and psychodyslpetics.

The most common symptom at admission was psychomotor agitation, which was observed in 43 patients, followed by reference ideation (n = 36) and paranoid delusional ideation (n = 30). Physical restraint was necessary for 14 patients (4%) in order to control symptoms (e.g., aggressiveness with concrete risk of harm to self or others). A detailed overview of symptoms detected upon evaluation in the ER is displayed in **Table 3**. The distribution of symptoms according to categories and specific groups of substances is highlighted in **Figure 1**.

**Table 3:** Symptoms observed at admission.

Entry Evaluation	n	%
Spatial disorientation	17	5
Temporal disorientation	23	7
Paranoid delusional ideation	30	9
Mystic delusional ideation	8	3
Reference delusional ideation	36	11
Grandeur delusional ideation	22	7
Mood elevation	21	7
Mood lowering	22	7
Psychomotor agitation	43	13
Sexual disinhibition	11	3
Anxiety and somatic anxiety	24	8
Aggressiveness towards others	24	8
Self-harm behaviour	14	4
Suicidality	25	8

**Figure 2:**
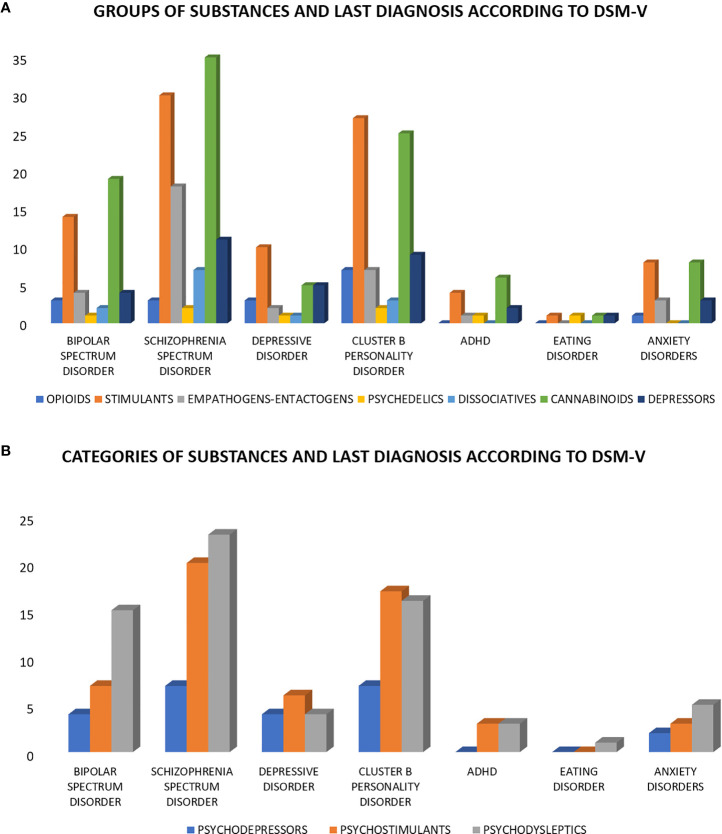
Categories and groups of substances and last diagnosis according to DSM-V. **(A)** Last DSM-V diagnosis distribution according to the following groups of substances: opioids, stimulants, empathogens-entactogens, psychedelics, dissociatives, cannabinoids, and depressors. **(B)** Last DSM-V diagnosis distribution according to the following categories of substances: psychodepressors, psychostimulants, and psychodyslpetics.

A large majority of patients in our sample (83%, n = 91) reported to have a previous psychiatric history: 31% had received only one DSM-5 diagnosis, 30% had received two, 22% had received three, 14% had received four and 3% had received more than four. Among those who had previously received only one diagnosis according to DSM-5 criteria, bipolar disorder (21%), and psychotic episode (32%) were the most common. The psychiatric diagnoses associated with each group and category of substances are shown in **Figure 2**.

We investigated possible associations between psychoactive substance use and specific psychiatric diagnoses. In particular, we found a positive association between psychoactive substance use and lifetime diagnosis of bipolar disorder (two-tailed Fisher’s exact test p = 0.013). Unsurprisingly, a positive correlation with a previous diagnosis of SUD was also reported (two-tailed Fisher’s exact test p = 0.003). No significant association between substance use and the other psychiatric diagnoses was found (two-tailed Fisher’s exact test p > 0.05).

Positive associations were also found with temporal disorientation at admission (two-tailed Fisher’s exact test p = 0.022), although data were available for only 63 patients. No significant association between substance use and any other symptom at admission was found (two-tailed Fisher’s exact test p > 0.05).

### Substance Use and Psychodiagnostics Scales

**Figure 3:**
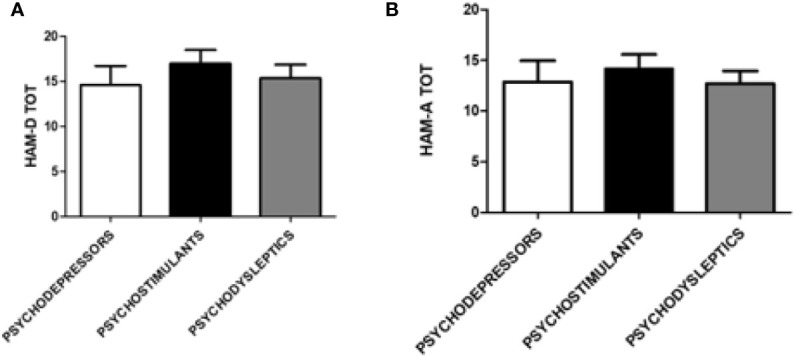
Hamilton Depression (HAM-D) and Anxiety (HAM-A total) scales scores according to categories of substances. **(A)** HAM-D scale score according to the following categories of substances: psychodepressors, psychostimulants, and psychodyslpetics. **(B)** HAM-A total scale score according to the following categories of substances: psychodepressors, psychostimulants, and psychodyslpetics.

Severity of anxiety and depression symptoms according to HAM-A and HAM-D scales were comparable among different substance categories. The distribution of scores on the aforementioned scales is available in **Figure 3**.

**Figure 4:**
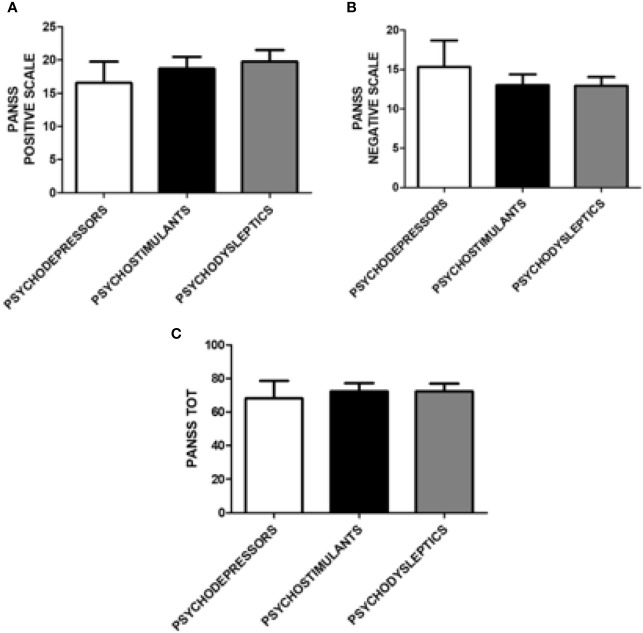
Positive and Negative Syndrome Scale (PANSS) positive, negative, and total scores according to categories of substances. **(A)** PANSS positive score according to the following categories of substances: psychodepressors, psychostimulants, and psychodyslpetics. **(B)** PANSS negative score according to the following categories of substances: psychodepressors, psychostimulants, and psychodyslpetics. **(C)** PANSS total score according to the following categories of substances: psychodepressors, psychostimulants, and psychodyslpetics.

Similarly, scores on the PANNS positive and negative subscales and total scores were equally distributed among categories of substances, as shown in **Figure 4**.

**Figure 5:**
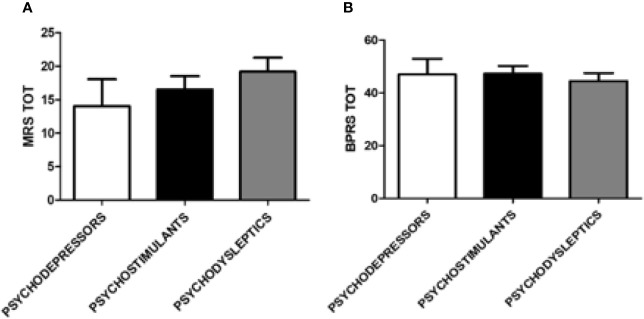
Mania Rating Scale (MRS) and Brief Psychiatric Rating Scale (BPRS) scores according to categories of substances. **(A)** MRS score according to the following categories of substances: psychodepressors, psychostimulants, and psychodyslpetics. **(B)** BPRS score according to the following categories of substances: psychodepressors, psychostimulants, and psychodyslpetics.

Presence and severity of mania and associated symptoms according to MRS were comparable among the different categories of substances. Finally, for general psychiatric symptoms assessed with the BPRS, no significant differences were found among depressor, stimulant and psychodysleptics users (**Figure 5**).

**Figure 6:**
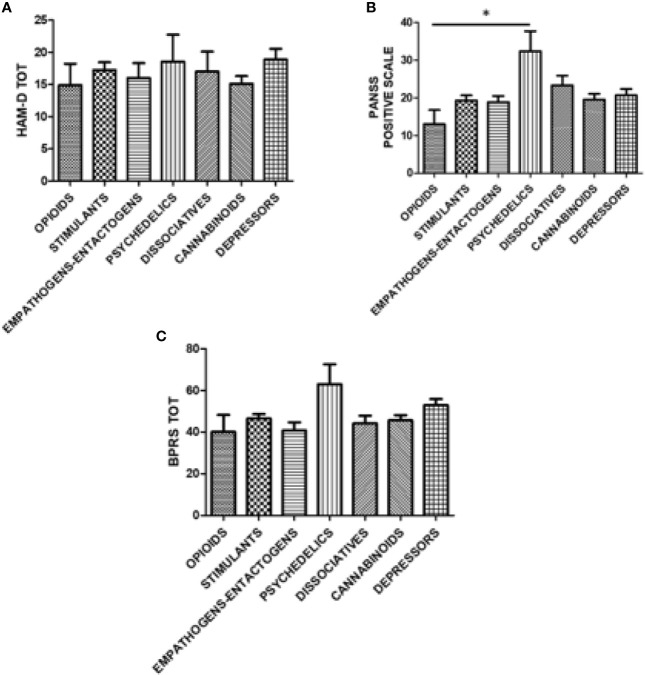
Hamilton Depression (HAM-D) Scale, Positive Syndrome Scale (PANSS), and Brief Psychiatric Rating Scale (BPRS) scores according to groups of substances. **(A)** HAM-D scale score according to the following groups of substances: opioids, stimulants, empathogens-entactogens, psychedelics, dissociatives, cannabinoids, and depressors. **(B)** PANSS positive score according to the following groups of substances: opioids, stimulants, empathogens-entactogens, psychedelics, dissociatives, cannabinoids, and depressors; *p < 0.05. **(C)** BPRS score according to the following groups of substances: opioids, stimulants, empathogens-entactogens, psychedelics, dissociatives, cannabinoids, and depressors.

Severity of psychiatric symptoms according to HAM-D, HAM-A, BPRS, and MRS were comparable among specific groups of substances. However, it was possible to highlight a higher prevalence of psychotic symptoms, according to the PANSS Positive Scale, for psychedelic substances users compared to opioid users (**Figure 6B**).

Results of the linear regression analysis highlighted a weak positive correlation between psychedelics use and PANNS Positive Symptoms scores (R = 0.374) and BPRS scores (R = 0.324), as well as between depressors use and BPRS scores (R = 0.322).

### Diagnoses Recorded at Discharge

Among the 110 participants, the most common psychiatric diagnosis at discharge was SUD (n = 44), followed by psychotic episode (n = 29). The main diagnoses assigned to patients after dismission from the psychiatric ward are described in **Table 4**.

**Table 4:** Discharge diagnoses.

Discharge Diagnosis	N	%
Bipolar disorder	9	6
Psychotic episode	29	19
Cluster B personality disorder	13	8
Substance use disorder	44	28
Alcohol abuse	16	10
Paranoid schizophrenia	4	2
Schizoaffective disorder	1	1
Manic Episode	9	6
Depression	6	4
Behavior Disorder	18	12
Adjustment Disorder	3	2
Anxiety Disorder	3	2

## Discussion

In this study, we described the acute psychiatric presentations related to recreational drug use in an adult population of subjects on holiday in Ibiza. This region has the highest number of nights spent by EU residents in tourist accommodation establishments (34) and has a flourishing “substance market”, which is frequently updated with newly developed recreational drugs. The characteristics of our sample, with high levels of education and good employment rates, differ from the typical profile of a substance abuser (35). An explanation for this phenomenon might be that the characteristics of substance-using clients have changed over recent years. In particular, clubbers and recreational drug users differ greatly from the “drug addicts” of the past (3, 36, 37). Moreover, Ibiza is a peculiar scenario in which subjects from low-income classes may not be able to find affordable facilities, while on the other hand, young tourists choose to spend most of their money earned during the winter period.

Cannabis and cocaine (in its different formulations, including crack) reported by users in the TLFB and by the urinalysis, were the most commonly referred substances, along with the presence of other traditional substances such as opiates, psychedelics, and entactogens. Alcohol was also commonly reported, mainly in association with other molecules. These findings have been confirmed by other studies that have demonstrated that most common emergency presentations related to acute recreational drug toxicity were associated with cocaine and cannabis use (38). NPSs represent a limited proportion (20%) of those reported. This data is of some interest, given that this is a real-life sample composed mostly of holidaymakers, among whom “2.0 online psychonauts” (39) do not represent the classical phenotype. Polysubstance abuse, including the use of two to six different substances during the same night, with alcohol as the substance most involved, followed by cannabis and cocaine, was showed to be a common behavior. The combination of psychoactive drugs may have numerous health implications (33, 40) and has been linked to increased levels of intoxication and possible fatality (41). Therefore, although a main “preferred” substance could be frequently identified with the structured interview and urinalysis data, the presence of polysubstance abuse appeared to be the relative norm.

Regarding the psychopathological evaluation upon admission, it is interesting to note the presence of spatial and temporal disorganization—an indication of qualitative alteration of consciousness. This phenomenon was evidenced mainly among users of psychostimulants and psychodysleptics. These types of manifestations are typical of the twilight state already described in SUDs (42) and are characterized by a state of clouded consciousness in which the individual is temporarily unaware of his or her surroundings. Our study confirms the importance of a correct assessment of the patient’s state of consciousness. These transient cognitive disorders are quite unusual in psychiatric illnesses, with the exception of dissociative disorders, frequently described in the literature as substance-induced symptoms (43). The qualitative alterations of consciousness could, in fact, be characterized as distinguishing elements between a classic psychotic episode and an episode induced by substances. Moreover, it could become a predisposing factor facilitating the development of delusional thoughts. Among the other phenomena found in the initial evaluation of the patients, the presence of psychomotor agitation, aggression, self-harm and suicidality stand out, specifically in the group of psychodysleptics and psychostimulant users. This data still emphasizes that this type of patient represents a category at risk, also in terms of public health, as recently reported in other studies (44, 45). Reference and paranoid delusions were the most commonly described delusional phenomena, specifically in the category of psychostimulants and psychodysleptics users, in accordance with the literature (46). The substance- or alcohol-induced delirium is often characterized by confirmation and interpretation, rather than by revelation, and by imaginative contents. The delusions reported are similar to paraphrenic delusions, with a feeling of unreality, while the ability to analyze the feeling is preserved. In this regard, the model of the Lysergic Psychoma could be an interesting proposal (14). As already highlighted by previous studies (47), relevant symptoms were also inherent to the sphere of affectivity, both in the direction of positive and negative polarity. This data confirms how substance-induced phenomena often have a significant affective component within them, probably deriving from the stimulation of both the dopaminergic and serotonergic systems (48).

Among the psychiatric manifestations observed in our sample, psychotic and mood symptoms predominated. Our findings have been confirmed by Acciavatti et al., who evidenced bipolar disorder and schizophrenia as the main psychiatric diagnostic frameworks within which the use of psychoactive substances is reported (47). Specifically, our study showed a significant association between a previous diagnosis of bipolar disorder and the use of substances. As regards the presence of the different diagnostic frameworks according to DSM-5 and the different types of substances, it should be emphasized that in the group of subjects with a diagnosis of schizophrenic spectrum disorder, a substantial number of subjects reported the use of entactogens/empathogens substances and dissociatives. This issue should be further investigated in future studies, given its relevance in terms of possible preventive strategies. Other diagnostic frameworks of greater response were those of depressive disorders, widely distributed among users of depressors and opiates, that of cluster B personality disorders, anxiety disorders, ADHD and eating behavior disorders. These data are in agreement with other studies in the literature with similar cases of substance abuse patients (49–51).

Mean scores of psychodiagnostic scales showed that psychiatric manifestations linked to psychoactive substances are characterized by a globally high level of psychopathology. As expected, PANSS Positive mean scores were higher than PANSS Negative mean scores, which is consistent with previous reports: a 2016 study demonstrated that patients with substance-induced psychosis had similar PANSS positive and significantly lower PANSS negative scores than patients with schizophrenia (4, 52). This data shows that the psychopathological potential of recreational substances is considerable, also in subjects without previous psychiatric symptoms of clinical relevance. We hypothesized that specific high potency substances, considerable amounts of a substance, and a high frequency of use may represent trigger factors. By differentiating between the groups of substances, it was possible to highlight how the use of psychedelics, compared to that of opiates, was more strongly associated with the presence of positive symptoms for PANSS. This data confirms that opiates can have an antipsychotic potential, or in any case, represent a group of substances with a low potential to induce positive symptoms (53).

In relation to the results for the other psychopathological scales of depression, anxiety, and mania, there is also evidence of high score levels. However, no significant differences are observed between the different groups (Opioids, Stimulants, Empathogens-Entactogens, Psychedelics, Dissociatives, Cannabinoids, and Depressors) or between the different categories of substances (Psychostimulants, Psychodysleptics, and Psychodepressors). This result could be explained by the high prevalence of poly-abuse, described in the majority of patients evaluated and also involving more than four substances together during the same night. For this reason, it is credible that possible intrinsic differences, with respect to the mechanism of action of the individual substances, have not resulted in fully manifesting themselves and, therefore, have not reached specific significance levels. If, on the one hand, this result may appear unsatisfactory, on the other, it should be emphasized that it represents a real-life population of abusers, in which the presence of a mono-substance user, always faithful to a single type of drug, represents an ideal scenario. This may also represent an important groundwork for harm reduction and prevention policies.

It is interesting to note the disparity between the diagnoses at discharge and the acute symptoms that have been observed upon admission. This data demonstrates how the psychoactive substance-induced phenomena often act as confounding factors for a correct diagnosis. At discharge from the hospital, the diagnosis of psychotic episode was reported in more than half of the participants. However, such a diagnosis is of an unclear nature, mostly in terms of future developments. Another relevant area of diagnosis was that of mood disorders (54% of the sample), with manic episodes in a high percentage of cases. Also, at discharge, multiple diagnoses still coexist, confirming the complexity of the field.

### Limitations of the Study

This study presents limitations: 1) the possibility to identify new substances in urine samples remains both complex and limited, which also applies to post-mortem samples. We performed a professional urine drug screening but the comparison between the self-report and objective data is still far from being considered reliable; 2) in our analysis, we did not consider the intoxication cases managed at the emergency department and not admitted to the psychiatry unit. With this bias, we have probably excluded from our evaluation those subjects with acute and rapidly transient psychiatric reactions; 3) the long-term effects of novel and traditional substances are still a matter of discussion and are difficult to assess without follow-up examinations; and 4) differentiation according to groups (Opioids, Stimulants, Empathogens-Entactogens, Psychedelics, Dissociatives, Cannabinoids, and Depressors) and categories (Psychostimulants, Psychodysleptics, and Psychodepressors) is probably not ideal, as multiple substance abuse was the predominant behavior.

In future studies, the following points shall be addressed: 1) to better discriminate the psychopathological effects of specific substances, including NPS and select a common ground able to help in differential diagnosis, 2) to prospectively look at the long-term effects, 3) to retrospectively observe which pharmacological treatments show higher levels of effectiveness.

## Conclusions

In a sample of subjects admitted to a psychiatric ward in a nightlife resort area, the use of psychoactive substances was notable and characterized by poly-use of both traditional and novel substances, with a relevant number of complex psychopathological consequences. Positive and manic symptoms and aggressiveness, including self-harm and suicidality, were highly represented, as well as state-of-consciousness alterations. These symptoms were not always transient in their nature and sometimes difficult to categorize *via* psychiatric diagnoses built predominantly for subjects without current use of substances. All of the above-mentioned considerations should be investigated in further studies, together with careful monitoring of critical “hotspots” of substance misuse, in order to design better and targeted prevention strategies.

## Data Availability Statement

The original contributions presented in the study are included in the article/supplementary material; further inquiries can be directed to the corresponding author.

## Ethics Statement

Data collection was carried out in an anonymous and confidential way; all participants received a detailed explanation of the design of the study and written informed consent was systematically obtained from every subject, according to the Declaration of Helsinki. Ethics approval was granted by the University of Hertfordshire Health and Human Sciences ECDA, protocol no. aPHAEC1042(03); by the CEI Illes Balears, protocol no. IB 2561/15 PI; and by the University “G.d’Annunzio” of Chieti Pescara, no. 7/09 04-2015. Majorcan local ethics committee also gave approval to the study.

## Author Contributions

GM, MG, and FS organized the study. GM and AN wrote the paper. SS and LT made the statistics. All the other authors actively participated in patients recruitment and evaluation.

## Funding

This study was partly found by the European Project entitled “Analysis, Knowledge dissemination, Justice implementation and Special Testing of Novel Synthetic Opioids” - JUST-2017-AG-DRUG.

## Conflict of Interest

The authors declare that the research was conducted in the absence of any commercial or financial relationships that could be construed as a potential conflict of interest.

The reviewer, SC, declared a shared affiliation, though no collaboration, with one of the authors, FS, to the handling editor.
